# Pharmacological and Therapeutic Approaches in the Treatment of Epilepsy

**DOI:** 10.3390/biomedicines9050470

**Published:** 2021-04-25

**Authors:** Shampa Ghosh, Jitendra Kumar Sinha, Tarab Khan, Kuramkote Shivanna Devaraju, Prabhakar Singh, Kumar Vaibhav, Pankaj Gaur

**Affiliations:** 1 ICMR-National Institute of Nutrition (NIN), Tarnaka, Hyderabad 500007, India; g.shampa17@gmail.com; 2 Amity Institute of Neuropsychology and Neurosciences (AINN), Amity University UP, Noida 201303, India; tarabkhan55@gmail.com; 3 Department of Biochemistry, Karnatak University, Dharwad 580003, India; ksdevaraju@kud.ac.in; 4 Department of Anatomy, All India Institute of Medical Sciences (AIIMS), Ansari Nagar, New Delhi 110029, India; prabhakar.singh@aiims.edu; 5 Department of Neurosurgery, Medical College of Georgia, Augusta University, Augusta, GA 30912, USA; kvaibhav@augusta.edu; 6 Lombardi Comprehensive Cancer Center, Georgetown University Medical Center, Washington, DC 20007, USA

**Keywords:** anti-convulsants, anti-epileptic drugs, drug targets, epileptogenesis, non-communicable disease, seizures, transcriptional modifications, pseudo-resistance

## Abstract

Epilepsy affects around 50 million people across the globe and is the third most common chronic brain disorder. It is a non-communicable disease of the brain that affects people of all ages. It is accompanied by depression, anxiety, and substantially increased morbidity and mortality. A large number of third-generation anti-epileptic drugs are available, but they have multiple side-effects causing a decline in the quality of life. The inheritance and etiology of epilepsy are complex with multiple underlying genetic and epigenetic mechanisms. Different neurotransmitters play intricate functions to maintain the normal physiology of various neurons. If there is any dysregulation of neurotransmission due to aberrant transmitter levels or their receptor biology, it can result in seizures. In this review, we have discussed the roles played by various neurotransmitters and their receptors in the pathophysiology of epilepsy. Drug-resistant epilepsy (DRE) has remained one of the forefront areas of epilepsy research for a long time. Understanding the mechanisms underlying DRE is of utmost importance because of its high incidence rate among epilepsy patients and increased risks of psychosocial problems and premature death. Here we have enumerated various hypotheses of DRE. Further, we have discussed different non-conventional therapeutic strategies, including combination therapy and non-drug treatment. The recent studies supporting the modern approaches for the treatment of epilepsy have been deliberated with particular reference to the mTOR pathway, breakdown of the blood-brain barrier, and inflammatory pathways.

## 1. Introduction

Epilepsy is a group of chronic non-communicable neurological disorders categorized by spontaneous recurrent seizures [1,2]. These seizures result from episodes of abnormal electrical activity in the brain. The process by which epilepsy develops in an otherwise normal brain is called epileptogenesis. Epilepsy may result from a head injury, brain tumors, brain infections like meningitis or encephalitis, stroke, birth defects, and sometimes even altered levels of entities like blood sugar or sodium [3]. It is the third most common chronic neurological disorder. It is a life-shortening brain disorder that affects around 50 million people or 1% of the world population. Although it is found across the globe, 80% of epileptic patients live in low- and middle-income countries. Epilepsy is characterized by 15 different types of seizures and 30 types of epilepsy syndromes and is accompanied by substantial comorbidity, depression, increased mortality, and anxiety [4,5].

During the last 30 years, there has been a huge advancement in the treatment of many types of seizures due to the introduction of over 15 third-generation anti-epileptic drugs (AEDs) [6]. Around 70–80% of patients enter remission with present AEDs who have new onset of epilepsy. Among 20–30% of patients, these medications are not able to control seizures. Besides, there is no AED that can prevent the development of the disease before the occurrence of the first seizure. Unfortunately, there is also drug-resistant epilepsy that is not controlled by or responds to AEDs. This shows the urgent need to develop appropriate therapeutic strategies to tackle the complex situation of epilepsy. Devising better treatment paradigms for epilepsy using various pharmaceutical and therapeutic approaches would need a better understanding of the different clinical and experimental strategies for the development and discovery of more efficient treatment methods that can help prevent and control the diseases [7,8,9].

## 2. Role of Genes, Genetics and Inheritance

The newly emerging genetic technology has played a significant role in the discovery of a variety of genes that are associated with epilepsy. A list of the few genes related to epilepsy is summarized in Table 1. The advancement of genomic techniques and gene sequencing has substantially enhanced the knowledge about the genetic variations taking place in the human genome. Studies estimate that there is an underlying genetic cause in about half of all cases of epilepsy [10]. Currently, with the emerging research of epigenetic biomarkers, MicroRNAs (miRNAs) have been assumed to play a significant role. MiRNA molecules 19-25 nucleotides long regulate gene expression as post-transcriptional modifiers [11]. Differential expression of more than 100 miRNAs has been reported in epilepsy. Among them, because of their predominant role in biological processes related to epilepsy, such as neurodegeneration, neuronal growth, and neuroinflammation, miR-132, miR-155, and miR-146a have been highlighted primarily [11]. Some cases of genetic mutations result in the core symptom of epilepsy, while changes in a few of the genes are responsible for malformations in the gross development of the brain that cause seizures. There are around 84 genes classified as epilepsy genes based on the OMIM database results. Mutation in these classified genes leads to epilepsy as a core symptom. There are approximately 73 genes that are categorized as neurodevelopment-associated epilepsy genes [10]. Twenty-four genetic variants have been identified by two large genome-wide association studies that are linked with epilepsy [12]. The defect in the *FMR1* gene is responsible for causing Fragile X Syndrome. Fragile X syndrome is one of the abnormalities associated with epilepsy and is characterized by intellectual dysfunction and abnormal behavior. According to the OMIM database (https://www.ncbi.nlm.nih.gov/omim, accessed on 24 April 2021), there are about 536 genes responsible for causing associated diseases of epilepsy [10,13].

One crucial feature of epilepsy is cognitive dysfunction. Deregulation of cognitive dysfunction is linked to activity-dependent transcription, which is essential for the transition of neuronal plasticity to long-term from short-term [9]. Therefore, it has been put forward that long-term memory formation, which necessitates activation of transcriptional programs for various processes such as learning, depends on the chromatin modifications of activated neurons [14]. Early infantile epileptic encephalopathy (EIEE) is marked by the presence of intellect deficits and seizures. The X-linked diseases are responsible for exhibiting many severe phenotypes in males as compared to females. However, epilepsy caused by mutations in protocadherin 19 (PCDH19) causes epilepsy in females that are heterozygous but not in males that are hemizygous [15]. Protocadherins (PCDHs) play an essential role in various neurological processes such as synaptogenesis, axon guidance, etc. They are considered as the most prominent family of cell-cell adhesion molecules [16]. The genes that encode for protocadherins are present throughout the genome and are responsible for demonstrating an overlapping and exclusive pattern of expression in the mature brain and also during the development of CNS in several populations of neurons. The mechanism that underlies this X-linked pattern of inheritance of epilepsy is not known [15]. Researchers are working to decipher the basic mechanisms as well as to find appropriate therapeutic interventions.

## 3. Epigenetics Involved in Epilepsy

Recent studies show epigenetics playing essential roles in temporal lobe epilepsy [17]. Therefore, studying the role of epigenetic changes in the development of the disease has become an emerging topic in the area of research. The knowledge of epigenetic mechanisms helps in providing the putative conceptual framework in the development of therapies that can help in the prevention of the disease. Epileptogenesis should be considered as a target point for developing therapy when there is an increment in the severity and frequency of impulsive recurrent seizures. Various processes that take place along with epileptogenesis are mossy fiber sprouting, dysfunction of adenosine together with gliosis, aberrant connectivity, neuronal cell loss, and neuroinflammation [18,19]. TCF4, MECP2, UBE3A, and CHD2 are some of the regulatory genes associated with epilepsy. Among these, the CHD2 gene is responsible for encoding a protein that remodels chromatin, and deregulation of CHD2 might have a downstream effect on other genes [20].

Epigenetic modifications are also responsible for many of the pathological changes that take place during epileptogenesis. Many changes have been shown to occur in the central nervous system cells that alter the gene expression due to DNA methylation and histone acetylation and methylation [12,21]. Moreover, these changes happen quickly and regularly. DNA methylation-reliant alterations in the process of transcription of genes get induced by even a single change in neural synchronization, which ultimately induces a cascade of transcription factors leading to long-term alterations [21]. When DNA methylation was deliberated globally with the help of antibody capture and in the BRD2 gene promoter, variations in the lymphoblastoid cell lines were observed in epileptic patients. Furthermore, in the hippocampus of epileptic patients, alterations in DNA methylation were observed [12].

Various processes like histone modifications that involve either adding up or eliminating the acetyl or methyl groups are suggested to be associated with epileptogenesis. According to the hypothesis of DNA methylation implicated in epileptogenesis, seizures can induce epigenetic modifications and can exaggerate the process of epileptogenesis. DNA hypermethylation, along with the amplified activity of DNA methylating enzymes has been implicated in the development of experimental and human epilepsy [22]. During brain development, DNA methyltransferases (DNMTs) have been shown to perform a significant role and expressed more predominantly in neurons than glial cells. Moreover, in the adult brain, studies have exhibited the functional significance of DNMTs in processes such as memory, learning, behavior, and synaptic plasticity. Thus, DNA methylation is speculated in mediating the process of epileptogenesis by leaving an imprint on physiological processes and gene expression, which might lead to the development of temporal lobe epilepsy later in life [23,24].

Histone modification is one of the epigenetic mechanisms that have the considerable potential to alter the neuronal expression of genes by exerting their additive effects in a correlated manner [17]. The principal role of histone proteins is to support the tertiary and quaternary structure of the DNA. Disruption in these vital epigenetic machinery leads to various disorders such as epilepsy, autism, Rett syndrome, etc. Epigenetic alterations that occur at the histone tail are responsible for influencing the structure of the chromatin that ultimately modifies the approachability of transcriptional regulators. One of the significant histone modifications that have been linked to epilepsy is lysine acetylation. In several animal models, the process of histone deacetylation by restraining histone deacetylase enzymes proved to be beneficial in preventing the symptoms of epilepsy [25].

DNA methylation is dependent on several biochemical enzymatic reactions. One such reaction pathway is the S-adenosylmethionine-dependent transmethylation pathway, which is controlled by glycine and adenosine under the regulation of adenosine kinase (ADK). In chronic epilepsy, it is observed that there is an increase in the ADK and a resulting decline in adenosine, which leads to elevated DNA methylation in the brain. Thus, interference with methylation of DNA gives the new conceptual prospect to control and prevent epilepsy. Glycine modifying therapies can also be regarded as an alternative opportunity that affects the process of DNA methylation and, ultimately, the process of epileptogenesis. Therefore, understanding the epigenetics of epileptogenesis might help in the discovery and development of therapeutic interventions [18,26]. Ketone bodies are found to play an important role of signaling molecules as well in addition to be a source of energy. Class 1 histone deacetylases (HDACs) are inhibited by β–hydroxybutyrate (BHB), which is considered as a ketone body with signaling functions. HDACs are responsible for the subtraction of acetyl groups from histone tails. These epigenetic modifications by ketone bodies are believed to be regulating gene expression and other epigenome modifications, which can implicate the treatment of several human diseases like epilepsy [24,27,28].

## 4. Neurotransmitter Release Machinery in Epilepsy

### 4.1. Glutamate Receptors

**Figure 1:**
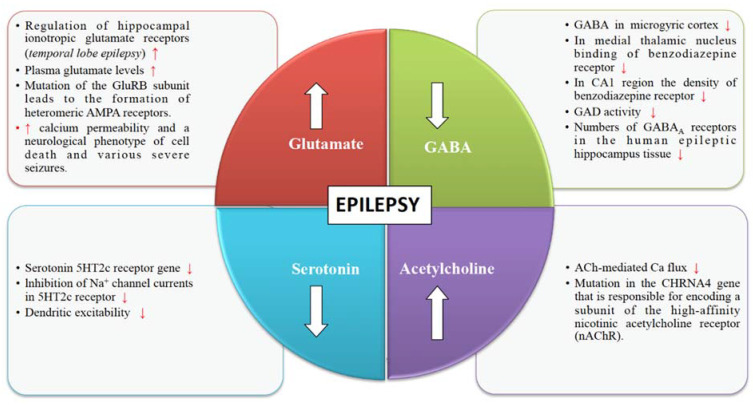
Neurotransmitter mediated changes in epilepsy. Different changes have been observed in the brain due to the increase in the levels of glutamate and acetylcholine and a decrease in the levels of serotonin and GABA. Such modulations due to various physiological conditions or comorbid situations have been reported, which should be taken into account while designing any pharmacological interventions in the epileptic patients.

Glutamate is an excitatory neurotransmitter responsible for stimulating an increase in calcium and sodium conduction through ligand-gated ion channels (Figure 1). A wide spectrum of anti-convulsant properties is displayed by AMPA antagonists and NMDA antagonists in animal models with acute and chronic epilepsy [29]. Once seizures begin, the activity-dependent plasticity of the glutamate receptors becomes a vital feature of the epileptic brain. Epileptogenesis has been linked to the receptor pore properties through the mechanism of mutations of the calcium-impermeable and AMPA-sensitive glutamate receptors. AMPA receptors in the central nervous system contain several subunits like GluR1, GluR2, GluR3, and GluR4. Mutation of the GluR2 (also known as GluA2 or GRIA2) subunit leads to the formation of heteromeric AMPA receptors, which lack the essential pore lining arginine site, which is responsible for conferring both calcium impermeability as well as single-channel conductance of three-fold attenuation. Mice bearing this mutation gather AMPA receptors with increased calcium permeability and a neurological phenotype of cell death and various severe seizures [30,31]. Additionally, glutamate, which is released at the synapse that acts on the metabotropic and ionotropic receptors, is responsible for the stimulation and escalation of the seizure activity [29]. The Na^+^-dependent transporters remove the glutamate from the extracellular space and synaptic cleft. Reduced expression of the glutamate transporters can induce seizures. Genetic manipulations related to the functioning of the glutamate receptor proteins in rodent models can enhance the threshold of seizures [32].

### 4.2. GABA Receptors

Various pieces of evidence from several clinical and experimental data underline the role of GABA in the mechanism and management of epilepsy (Figure 1). The synaptic inhibition by GABA plays an essential role in regulating neuronal excitability, which has been linked to epilepsy. GABAergic neurotransmission that is controlled by Cl^–^ permeable GABA_A_ receptors can exhibit both seizure-repressing and -stimulating activity [33]. GABAergic functions have shown abnormalities in the acquired and genetic animal models of epilepsy. Several studies have shown changes in the GABA receptor densities and concentrations of GABA in the human epileptic tissue. It has been found that there are reduced numbers of GABA_A_ receptors in the human epileptic hippocampus tissue [34]. Many researchers have shown that seizures are suppressed by GABA agonists and are produced by GABA antagonists. GABA agonists are anticonvulsants whereas GABA antagonists are proconvulsant. Seizures are caused by drugs that inhibit the synthesis of GABA. Some of the GABA synthesis inhibitors include L-allyglycine, isoniazid, thiosemicarbazide, and 4-deoxypyridoxine. Therefore, inhibition of the synthesis of GABA is known to be epileptogenic [34]. Effective anti-convulsants like barbiturates and benzodiazepines (BZDs) function by increasing the GABA mediated inhibition. Barbiturates are responsible for extending the opening time of the chloride channels. Benzodiazapenes also amplify the chloride channel opening rate by enhancing the binding of the GABA to its receptors [35]. Drugs like vigabatrin and tiagabine are effective anti-convulsants that enhance synaptic GABA by decreasing GABA catabolism or reuptake, ultimately responsible for inhibiting seizure activity. Drugs that play a significant role in increasing synaptic GABA have been shown to be effective AEDs [36].

### 4.3. Cholinergic Receptors

The structural and functional diversity of the neuronal nicotinic acetylcholine receptors (nAChRs) perform modulatory functions throughout the mammalian brain (Figure 1). Nicotinic receptors are involved in various developmental mechanisms such as memory, attention, and learning. Disruptions in cholinergic mechanisms can lead to several disorders such as epilepsy, Parkinson’s disease, dementia, schizophrenia, autism, Alzheimer’s disease, etc. [37]. Functional nAChRs are broadly dispersed throughout the central nervous system and are situated on dendrites, axon terminals, and cell bodies and mediate synaptic neurotransmission [38]. According to genetic studies conducted in several animal models and epileptic patients, it has been illustrated that the activity of nAChRs is altered in certain types of epilepsy such as juvenile myoclonic epilepsy (JME) and autosomal dominant nocturnal frontal lobe epilepsy (ADNFLE) [39]. Cholinergic receptors are broadly dispersed on both inhibitory and excitatory interneurons present in and outside of the frontal lobe. Pentamers, which are assembled in the subunit pattern of (2α-3β), form the widely held nicotinic receptors of the brain. Five mutations in these two subunits have been related to autosomal dominant frontal lobe epilepsy [40]. Acetylcholinergic neurons play an essential role in the mental development in the pedunculopontine tegmental nucleus. Disturbance in the nAChRs can give rise to epilepsy [41].

### 4.4. Serotonin Receptors

Serotonergic neurotransmission has been shown to have a potential role in epilepsy [42]. Serotonin receptors (5-HTRs) have been therefore considered as promising candidate targets for the development of new AEDs (Figure 1). Studies show 5-HT regulates a wide variety of focal and generalized seizures. Agents like 5-hydroxytryptophan and 5-HT reuptake blockers are known to increase the extracellular serotonin levels and hence inhibit focal as well as generalized seizures. On the other hand, depletion of serotonin levels in the brain is known to subside the threshold for different evoked convulsions [43]. Selective serotonin reuptake inhibitors (SSRIs) can enhance the levels of serotonin at synapse but reduces the synthesis of the serotonin in the brain. Persistent administration of SSRIs are known to be one of the cause for the decreased synthesis of serotonin and increases seizure susceptibility [44]. Spontaneous seizures and a decreased threshold for audiogenic seizures have been linked to the targeted subtraction of the serotonin 5HT2c receptor gene. The 5HT2c receptor plays a significant role in modulating a persistent Na^+^ current. It is shown that in cortical neurons, 5-HT2a/c receptor activation is reduced via rapidly inactivating Na+ currents by reducing the maximal current amplitude and shifting fast inactivation voltage dependence. 5-HT2a/c receptor stimulation also reduced the amplitude of persistent Na+ current without altering its activation voltage dependence. This modulation is mediated by a PKC-dependent mechanism and further reduces the dendritic excitability. Seizure susceptibility may be enhanced by the loss of inhibition of Na+ channel currents in 5HT2c receptor [45,46].

## 5. Drug Resistant Epilepsy

Drug-resistant epilepsy (DRE) is also known as refractory epilepsy or pharmaco-resistant epilepsy. It can be defined as a failure of two or more sufficient trials of tolerated, chosen, and appropriately used AEDs regimens, which can be administered as monotherapies or in combination to get relief from seizures. Around one out of four patients with seizures develop DRE [47]. DRE patients have increased risks of injuries, psychosocial problems, and premature death [48,49,50]. Some of the hypothesized mechanisms underlying the cause of DRE are discussed below.

### 5.1. Alterations in the Drug Targets

This hypothesis states that sensitivity to the treatment is decreased due to the alterations in the cellular targets of the drugs. The α_2_ subunit of the neuronal Na^+^ channel, which is encoded by the SCN2A gene, has been found to be related to anti-epileptic drug resistance [47]. However, this hypothesis cannot explain the contributory role of the alterations in drug targets in causing epilepsy in patients resistant to several drugs with different modes of action [51,52].

### 5.2. The Inability of the Drugs to Reach Their Targets

This transporter hypothesis postulates that at the epileptic target, drug resistance may apply to the overexpression of the multidrug efflux transporters. P-glycoprotein is the most widely researched efflux transporter whose primary function is to maintain the integrity of the blood–brain barrier by decreasing the cerebral build-up of the substrate drugs [47]. Up-regulation of efflux transporters such as P-glycoprotein in capillaries and abnormal expression in neuronal and glial cells has been described in various studies on patients with DRE [53].

### 5.3. Real Targets Missed by the Drugs

Presently, the primary usage of the AEDs is only to prevent seizures rather than focusing on the pathogenic processes that are causing the disease. Autoantibodies to the ion channels that are associated with neuronal inhibition and excitation, including voltage-gated ion channels and NMDA and GABA receptors, have been identified in patients with seizures. Such cases have been seen predominantly in multiple circumstances of encephalitis and occult cancer. However, these patients usually are unable to respond to conventional anti-epileptic drugs [47,54,55].

## 6. Non-Conventional Therapeutic Strategies

The available AEDs control the generating tendency of seizures and are effective in about 60% of individuals. Additionally, most of the existing medications have adverse drug reactions and hypersensitivity issues. It further aggravates by the increased incidence of mental health problems, depression, anxiety, and suicidal tendencies [56]. In such intricate situations, one of the important ways to cope up is the management of the disorder through patient care and devising different therapeutic strategies.

### 6.1. Ruling Out Pseudo-Resistance

It is the phenomenon in which the seizures persevere because the fundamental disorder has not been adequately treated. So, it is imperative to rule out or correct the underlying disorder before the drug treatment can be considered to have failed. There can be several situations in which misdiagnosis of epilepsy can take place. Cardiac arrhythmia, vasovagal syncope, and other neurological disorders such as migraine and transient ischemic attacks are some of the conditions that imitate epileptic seizures [47,57]. The patient’s behavior and lifestyle can also be the possible cause of pseudo-resistance. Alcohol addiction, stress, sleep deprivation, and drug abuse are also the common factors that can cause seizures [58]. Therefore, it is essential to first rule out the chances of misdiagnosis before starting the medication paradigm or changing it once started.

### 6.2. Combination Therapy

According to many studies and research data, the combination of drugs helps in controlling the disease. The same drugs at differential dosages have been seen to suit different patients. This may be due to the fact that at any instance, separate pathophysiological mechanisms can occur in the same individual. Such a situation would warrant the usage of different therapeutic strategies at different levels for better results. Various natural or herbal-based drugs like Cicuta virosa and Nux vomica have been shown to be effective in reducing seizure activity and other physiological parameters in animal models of epilepsy [9,59]. Several animal models have supported evidence that a combination of lamotrigine and sodium valproate aids in the management of partial-onset and generalized seizures. Other usually recommended combinations include valproate with ethosuximide for controlling absence seizures and lamotrigine with topiramate for controlling a wide range of seizures [47,60].

### 6.3. Non-Drug Treatment

Patients who are suffering from DRE have surgery as an alternative method for treatment, mainly if they have a surgically remedial disease like unilateral hippocampal sclerosis or other curable lesions. Therefore, depending on the indication, a number of surgical procedures can be performed to treat and control epilepsy after the deliberation of further trials of anti-epileptic drugs [61]. In children with DRE, the ketogenic diet is used as a management strategy. These are also available as formula-based ketogenic diets for infants [47]. A ketogenic diet seems to be helpful in controlling many types of seizures. Through the epigenetic mechanism, a ketogenic diet is known to be responsible for regulating gene expression. It has been found that dietary intake of donors of methyl groups like choline can have an intense effect on the process of DNA methylation. Ketogenic diets with high-fat and low-carbohydrate content are observed to be highly useful in the treatment of refractory seizures in kids [24]. Additionally, various studies on the micronutrient deficiencies like vitamin B12 show possible involvement in the pathway and hence may prove to be beneficial in future therapeutic strategies [62,63]. In adults and adolescents, a device called the vagus nerve stimulator has been approved for use as an adjunctive therapy with partial-onset seizures that are resistant to anti-epileptic drugs [47,64]. A vagus nerve stimulator is used to generate a pulse and is implanted in the upper chest of the patient, which then provides electrical current to the vagus nerve of the neck [65].

## 7. Modern Approaches for Treatment

Approved AEDs for the treatment of epilepsy work by various mechanisms that mainly include the modulation of voltage-dependent ion channels, activation of GABA, and inhibition of glutamate receptors. Several capable pathways including shared ones with neurodegenerative disorders [66] and potential drug targets have been identified by many researchers and some of them are discussed here.

### 7.1. MTOR Pathway

This signaling pathway is the mammalian target of rapamycin, which is responsible for regulating cell growth, cell proliferation, cell differentiation, and cell metabolism in the brain. Many studies have shown that dysregulation of mTOR is responsible for the pathogenesis of acquired forms of epilepsy, such as temporal lobe epilepsy [6]. Therefore, therapeutic intervention in this pathway can lead to the discovery of more tolerable anti-epileptic and anti-epileptogenic drugs [67,68].

### 7.2. Inflammatory Pathways

Various studies and researches have shown shreds of evidence, which states that inflammatory mediators that are released by cells of the brain and peripheral immune cells are implicated in the foundation of seizures and the process of epileptogenesis [6]. Data and facts have emerged that changes in the inflammatory and immune pathways might be the consequences as well as the origin of the different types of epilepsy [69].

### 7.3. Breakdown of Blood-Brain Barrier

Irrespective of their etiology, dysfunction of the blood-brain barrier is a characteristic of epileptogenic injuries of the brain. Any harm to the blood-brain barrier microvasculature during the brain injury leads to the leakage of the serum albumin into the micro-environment of the cerebral cortex, which induces a signaling cascade in astrocytes, which results in local inflammation by activating the transforming growth factor β receptor (TGFβR) [70]. Dysfunction of the astrocytes leads to the impairment in the homeostasis of the extracellular brain environment, which further results in the increased excitability of the neurons. TGFβR is a remarkable novel target that interferes with the process of epileptogenesis. This is because of the obstruction of TGFβ signaling in the albumin is responsible for reversing the inflammation and transcriptional modifications linked with activated glia and thus prevents the progression of epileptogenesis [6,71]. Various approaches that are being employed in the therapeutics associated with epilepsy have been summarized in Table 2.

## 8. Conclusions

Several applicable models are available to study and understand the process of epileptogenesis [72]. However, there are still many challenges in dealing with epileptogenesis. There are so many emerging approaches that are under consideration for the treatment of DRE. Various researchers are working on pharmacotherapy and many other therapeutic approaches that can aid in the treatment of epilepsy. Complementary alternative medicines like Cicuta virosa [59] and Nux vomica [9] could be used in combination with lower doses of effective drugs for better results. Such newer combinations of drugs need more research to be done to find their efficacy. Many capable pathways including epigenomic maintenance through dietary intervention [73] and potential drug targets have been identified by many researchers that provide the approach for the treatment. The discovery of a wide variety of anti-epileptic drugs has provided great advancement in treating different types of seizures [72]. Further, understanding the role of different neurotransmitters and their effect on epileptogenesis can help in the emergence of novel treatment strategies for epilepsy in near future.

**Table 2:** Different approaches used for the treatment of epilepsy.

S. No.	Treatment Approaches	Interventions Used	Action Mechanism	Main Uses	References
**A.**	**PHARMACEUTICAL APPROACHES**	Gabapentin	Ca^2+^ blockage	Used for generalised and focal seizures.	[74,75]
	(Anti-epileptic drugs)	Carbamazepine	Na^+^ channel blockage	Decrease nerve impulses that are responsible for causing seizures.	[74]
		Lamotrigine	Na^+^ channel blockage	Used as a first- line drug for generalized and focal seizures.	[60,75]
		Tiagabine	GABA potentiation	Used for partial seizures in adjunctive therapy.	[74]
		Zonisamide	Na^+^ channel blockage	Used for generalized and focal seizures.	[74,75]
		Vigabatrin	GABA potentiation	Used for infantile spasms and for focal onset of seizures.	[74]
		Perampanel	Glutamate (AMPA) antagonist	Used for partial seizures with focal onset.	[74,75]
**B.**	**THERAPEUTIC APPROACHES**	Progressive muscle relaxation	Tense a group of muscles while breathing in and relaxes them while breathing out.	Improves sleep and overall well-being. Enhances control over epilepsy by the patients.	[76]
		Yoga	Release tension in key joints through combination of body postures.	Decrease in automatic dysfunction during onset of seizures.	[76,77]
		Cognitive behavioural therapy	Restructuring of maladaptive thought patterns.	Improvement in anxiety and depression and enhanced psychosocial functioning.	[76,78]
		Vagus nerve stimulation	Used to generate impulse through electric current in vagus nerve.	Used as an adjunctive therapy for partial onset of seizures.	[65]
**C.**	**NATURAL APPROACHES**	Ketogenic diet	Neurotransmitter modulation in brain by ketone bodies.	Successful in reducing seizures and enhancing motor function.	[27,64,79]
		Vitamin D3	Increase Ca^2+^ uptake and decrease neuronal excitability.	Produces anti-convulsant effect and prevent seizures.	[80,81]
		Herbal treatments	Found to be involved in potentiation of GABAergic activity in brain.	Herbal medications control epileptic seizures and reduce side effects and increase cognitive effects of AEDs.	[82,83]

## Figures and Tables

**Table 1:** List of genes associated with the pathophysiology of epilepsy. The pattern of inheritance, age of onset, and functional categories has been given in concurrence with the associated genes.

S.No.	Epilepsy Genes	Functional Category	Pattern of Inheritance	Type Of Syndrome	S.No.
1.	*ALDH7A1*	Enzyme	Autosomal recessive	Pyridoxine dependent epilepsy.	Neonatal period
2.	*KCNQ2*	Potassium channel	Autosomal dominant	Benign familial neonatal seizures.	
3.	*GABRA1*	Receptor of GABA A	Autosomal dominant	Early infantile epileptic encephalopathy.	Infancy and early childhood
4.	*SCN8A,* *SCN2A*	Sodium channel	Autosomal dominant	Benign familial neonatal seizures. Early infantile epileptic encephalopathy.	
5.	*CHD2*	Enzyme	Autosomal dominant	Childhood onset epileptic encephalopathy.	
6.	*STX1B*	Transport across membrane	Autosomal dominant	Generalized epilepsy.	
7.	*TBC1D24*	Modulator of enzyme	Autosomal recessive	Familial infantile myoclinic epilepsy. Early infantile epileptic encephalopathy.	
8.	*NECAP1*	Not classified	Autosomal recessive	Early infantile epileptic encephalopathy.	
9.	*UBA5,* *GNAO1*	Enzyme	Not known	Early infantile epileptic encephalopathy.	
	*HCN1*	HCN channel			
10.	*GPR98*	Receptor	Autosomal dominant	Familial febrile seizures.	
11.	*KCNMA1*	Potassium channel	Autosomal dominant	Generalized epilepsy with paroxysmal dyskinesia.	
12.	*STRGAL3,* *WWOX*	Enzyme	Autosomal recessive	Early infantile epileptic encephalopathy.	
13.	*PRRT2*	Not classified	Autosomal dominant	Benign familial infantile seizures.	
14.	*SLC6A1*	Transporter	Autosomal dominant	Myoclinic atonic epilepsy.	
15.	*ARHGEF9*	Modulator of enzyme	X linked recessive	Early infantile epileptic encephalopathy.	
16.	*SCN9A*	Sodium channel	Autosomal dominant	Dravet Syndrome. Familial febrile seizures.	
17.	*CDKL5*	Enzyme	X linked dominant	Early infantile epileptic encephalopathy.	
18.	*GRIN2A*	NMDA receptor	Autosomal dominant	Focal epilepsy with speech disorder.	
19.	*STRGAL5*	Enzyme	Autosomal recessive	Amish infantile epilepsy.	
20.	*CACNA1H*	Calcium channel	Not known	Childhood absence epilepsy.Idiopathic generalized epilepsy.	
21.	*ALG13*	Enzyme	X linked	Early infantile epileptic encephalopathy.	
22.	*CPA6*	Enzyme	Autosomal dominant	Familial temporal lobe epilepsy.	Juvenile phase and later
	*LGI1*	Not classified			
23.	*CACNB4*	Calcium channel	Autosomal dominant	Juvenile myoclinic epilepsy. Idiopathic generalized epilepsy.	
24.	*EFHC1*	Signalling molecule	Autosomal dominant	Juvenile absence epilepsy. Juvenile myoclinic epilepsy.	
25.	*CLCN2*	Chloride channel	Autosomal dominant	Juvenile generalized epilepsy. Juvenile absence epilepsy. Juvenile myoclinic epilepsy.	
26.	*ADRA2B*	Receptor	Autosomal dominant	Familial adult myoclinic epilepsy.	
27.	*GABRD*	Receptor of GABA A	Autosomal dominant	Generalized epilepsy with febrile seizures. Juvenile myoclinic epilepsy.	
28.	*CASR*	Receptor	Not known	Idiopathic generalized epilepsy.	
29.	*DEPDC5*	Not classified	Autosomal dominant	Familial focal epilepsy.	Unspecified
30.	*CHRNB2*	Acetylcholine receptor	Unknown	Nocturnal frontal lobe epilepsy.	
31.	*KCNC1*	Potassium channel	Autosomal dominant	Progressive myoclinic epilepsy.	
32.	*GOSR2*	Transport across membrane	Autosomal recessive	Progressive myoclinic epilepsy.	
	*CERS1*	Enzyme			
	*LMNB2*	Protein for cytoskeleton			
	*KCTD7*	Not classified			

## Data Availability

Not applicable.
